# Pitfalls in the Diagnosis of Anaplastic Large Cell Lymphoma with a Small Cell Pattern

**DOI:** 10.1155/2013/840253

**Published:** 2013-10-10

**Authors:** Rowan L. Grigoropoulos, Penny Wright, Mars B. van t'Veer, Mike A. Scott, George A. Follows

**Affiliations:** ^1^Department of Haematology, Cambridge University Hospitals NHS Foundation Trust, Hills Road, Cambridge CB20QQ, UK; ^2^Department of Histopathology, Cambridge University Hospitals NHS Foundation Trust, Hills Road, Cambridge CB20QQ, UK

## Abstract

Anaplastic large cell lymphoma with a small cell pattern is a rare T-cell lymphoma. This condition is more frequently seen in younger patients and should be considered when patients present with leucocytosis and constitutional symptoms. In this report, we describe our diagnostic work-up for one such case using blood, lymph node, and bone marrow aspirate samples, highlighting the variability of antigen expression seen in different sample types and methodologies. This case shows the importance of having a high index of suspicion and assessing CD30 and anaplastic lymphoma kinase expression in all suspected T-cell neoplasms even though this rare condition is not necessarily expected.

## 1. Introduction

Anaplastic large cell lymphoma (ALCL) anaplastic lymphoma kinase positive (ALK+) is one of the subcategories of the mature T-cell neoplasms [1]. ALCL, ALK+ accounts for approximately 3% of cases of adult non-Hodgkin lymphomas and 10–20% of childhood lymphomas. There are many morphological variants of ALCL of which the small cell pattern variant accounts for 5–10% of cases. In this rare subtype, leukaemic manifestation is commonly seen in contrast to the other morphological variants [1–3].

Clonal T-cell populations may be demonstrated in the peripheral blood in a variety of clinical conditions. Although lymphocyte morphology may be indicative of a specific pathology, phenotyping of the population of interest is valuable in establishing the diagnosis. The apparent phenotype however may vary depending on technique, sample type, and cell population analysed. It is therefore essential to correlate phenotypic information with genetics and clinical presentation.

## 2. Case Presentation

A 22-year-old female presented to her local hospital with a 16-day history of fevers, night sweats, nausea, and right upper quadrant abdominal pain. She also reported weight loss of nine and a half kilos. She had been referred several times to hospital and received several courses of intravenous antibiotics with no improvement. 

**Figure 1:**
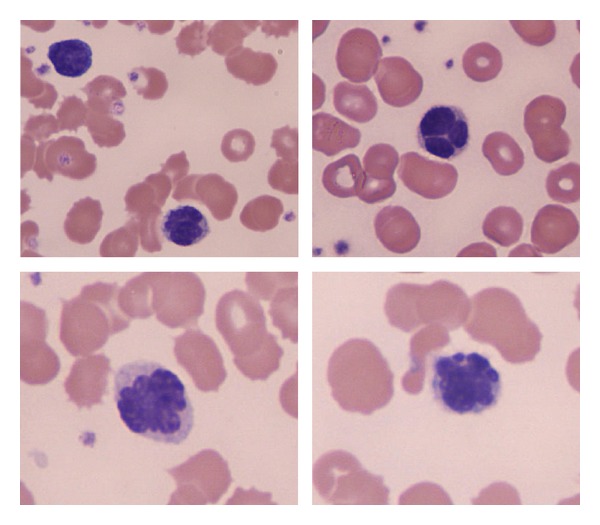
Peripheral blood smear, modified Wrights stain (original magnification ×1000) showing atypical lymphocytes with convoluted nuclei.

Full blood count analysis showed a leucocyte count of 14.2 × 10^9^/L with mild lymphocytosis (5.4 × 10^9^/L) and normal haemoglobin and platelet counts. A blood film showed a population of small, mature appearing lymphocytes with coarse nuclear chromatin and a second population of small lymphocytes with convoluted nuclei (Figure 1).

Biochemistry showed slightly elevated alanine transaminase (57 U/L (0–50 U/L)) and alkaline phosphatase (315 U/L (30–135 U/L)). Other parameters, including lactate dehydrogenase and beta-2-microglobulin, were normal.

Ultrasound showed splenomegaly (17.1 cm), and CT scans of the neck, chest, abdomen, and pelvis were performed. These showed significantly enlarged left axillary lymph nodes (largest 1.6 cm) as well as hilar, retrocrural, celiac, left gastric, and preaortic lymphadenopathy. 

The left axillary lymph node was excised and a blood sample was referred for immunophenotyping.

### 2.1. Immunophenotyping of the Peripheral Blood

**Figure 2:**
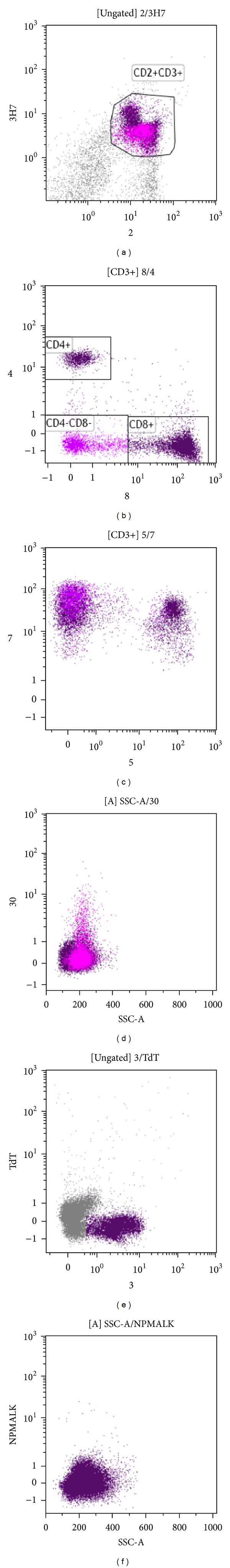
Flow cytometry of the peripheral blood showing T cells (coloured purple) identified by CD2/CD3 coexpression (a) subcategorised based on CD4 and CD8 expression with the CD4/CD8 “double-negative cells” coloured pink (b) showing CD7 and CD5 expression (c), CD30 expression (d), TdT (e) and NPM-ALK (f). Images compiled using Kaluza Flow Analysis software v1.2 from Beckman Coulter Inc.

Flow cytometry was performed using the whole blood lysis method and showed a mild CD8-positive T-cell lymphocytosis (3.1 × 10^9^/L) with normal expression of CD2, CD3, CD5, and CD7 and a population of CD4/CD8 “double-negative” T cells (1.2 × 10^9^/L). The CD4/CD8 double-negative T cells expressed CD2, weak CD3, and strong CD7. They were negative for CD4, CD5, CD8, CD10, CD30, HLA-DR, and TdT. NPM-ALK by flow cytometry was shown to be negative (method as described previously) [4] (Figures 2(a)–2(f)). The absence of CD30 expression was confirmed by APAAP staining of a peripheral blood smear.

### 2.2. Histopathology of the Left Axillary Lymph Node

**Figure 3:**
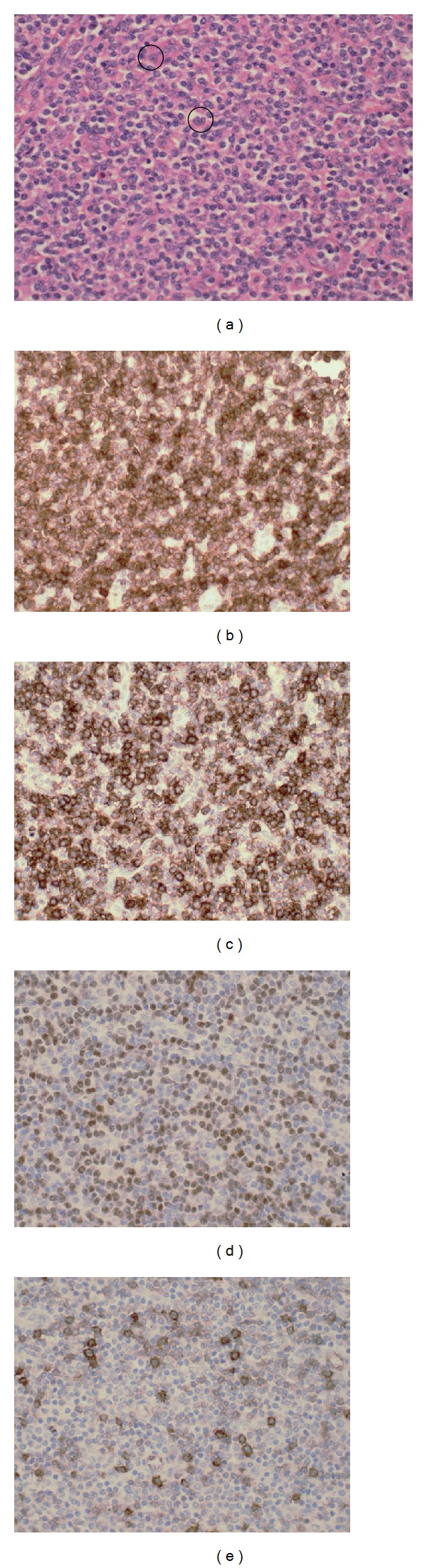
Left axillary lymph node biopsy (original magnification ×400). Haematoxylin and eosin: (a) infiltration of the node by predominantly small lymphoid cells and occasional “hallmark” cells (circled in black), CD3 immunostain positive (b), CD8 immunostain positive (c), ALK immunostain-positive nuclear staining (d), and CD30 immunostain scattered positivity in large cells (e).

Biopsy of a left axillary lymph node revealed diffuse effacement of the lymph node architecture by small-sized cells with irregular nuclear outlines, coarse chromatin, small nucleoli, and moderate amounts of eosinophilic cytoplasm. These cells infiltrated into the surrounding adipose tissue. Scattered larger cells with reniform nuclei were noted (Figure 3(a)).

Immunohistochemistry (IHC) showed the smaller cells to express CD2, CD3, CD7, and CD45 and have consistent nuclear confined expression of ALK. They did not express CD5, TdT, CD56, EMA, CD20, PAX 5, or CD10. Many of these were CD8 positive, but a population negative for CD4/CD8 was also described (Figures 3(b)–3(d)).

The scattered large cells showed variable expression of CD30 (Figure 3(e)). Perivascular location of these was noted very focally in the extranodal adipose tissue. 

### 2.3. Fluorescence In Situ Hybridisation (FISH) and Molecular Analysis of the Peripheral Blood and Lymph Node Biopsy

**Figure 4:**
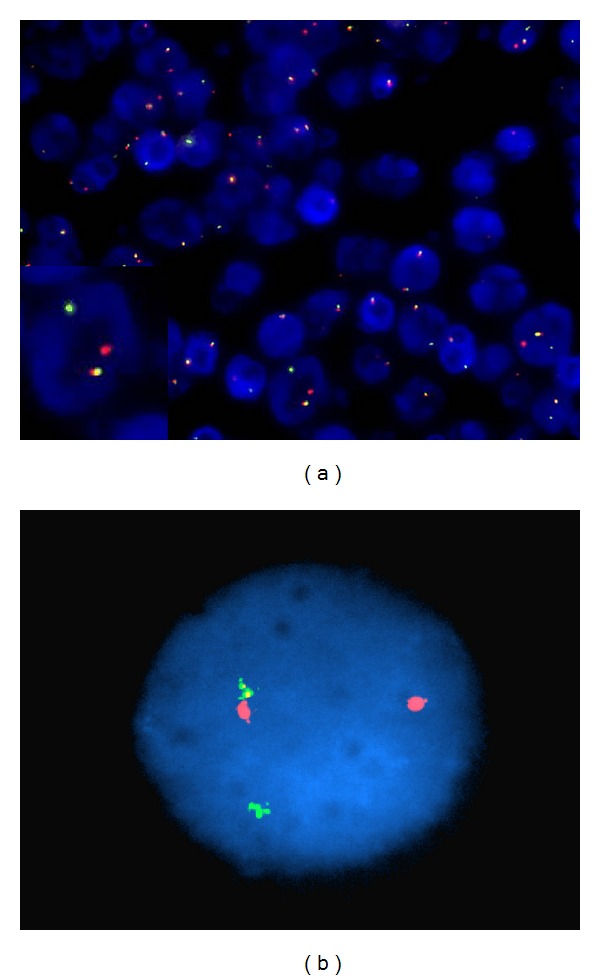
FISH analysis using ALK dual colour break-apart rearrangement probe showing breakage of the one allele of the ALK gene locus in many of the nuclei examined in left axillary lymph node biopsy, inset showing enlarged nucleus (original magnification ×200) (a) and the same findings in the peripheral blood (original magnification ×1000) (b).

Interphase FISH was performed on paraffin sections using a break-apart rearrangement probe and showed breakage of one allele of the ALK gene locus in the majority of cells examined (Figure 4(a)), indicating the presence of an ALK gene rearrangement, and these findings were also demonstrable in the peripheral blood (Figure 4(b)). 

Polymerase chain reaction (PCR) for TCR gene rearrangement showed clonal patterns for TRG@ and TRB@ in the peripheral blood consistent with a diagnosis of a T-cell lymphoproliferative disorder. Retrospective analysis of the peripheral blood demonstrated the NPM-ALK fusion transcript resulting from the *t*(2; 5) by real-time PCR.

A staging bone marrow showed a lymphocytic infiltrate of approximately 10% expressing CD2, CD3, and CD7, with many cells expressing CD8, weak CD30, and weak ALK.

### 2.4. Diagnosis and Treatment

Based on the morphology, variable CD30, nuclear confined ALK expression, and leukaemic presentation in a young patient, a diagnosis of ALK-positive anaplastic large cell lymphoma with a small cell pattern was made.

 The patient went on to receive six cycles of cyclophosphamide, doxorubicin, vincristine, and prednisolone (CHOP) and reached complete remission (eighteen months at the time of report).

## 3. Discussion

Leukaemic presentation in other morphological variants of ALCL is uncommon. Contrarily almost all cases with the rare small cell pattern present in this manner with leucocytosis of variable degree in both neutrophil and lymphocyte lineages. Suspicion should be raised in younger patients with these features and constitutional symptoms [2, 3]. 

ALCL with a small cell pattern in younger patients may be mistaken for an infective or inflammatory process and treatment with antibiotics in the weeks prior to diagnosis is not uncommon [3, 5]. Other frequently described features include hepatomegaly, splenomegaly, pleural effusions, weight loss, peripheral lymphadenopathy, and skin involvement [2].

 The peripheral blood often contains a population of small-medium sized lymphocytes with dense and lobulated nuclei. Rare large cells with basophilic, vacuolated cytoplasm may be present [6, 7]. Flow cytometry of these small cells typically shows a mature T-cell phenotype with loss of antigen expression being well described. CD30 and ALK are usually negative [2, 5]. T-cell clonality analysis of the peripheral blood can confirm clonally rearranged TCR *γ* and *β* genes in 90% and could also be used to detect *t*(2; 5) if suspected. In our case, a large cell population was not evident in the blood, and from the morphology and phenotype a diagnosis of ALCL was not suspected.

Diagnosis is predominantly made on lymph node histology which typically shows disruption to normal architecture by small-, medium-, and large-sized cells. These cells are usually positive for one or more T-cell antigens with the small cells often staining more intensely. The smaller cells are typically weak/negative for key diagnostic antigens CD30 and ALK, and it is likely that these cells are representative of those seen in the circulation. The larger reniform “hallmark” cells may be present singly or in clusters and often surround small blood vessels and sinuses. These show strong staining for CD30 which is generally confined to the cell membrane and Golgi regions. They are also strongly positive for ALK which in this small cell variant is often nuclear restricted [3, 5, 8, 9]. 

Staging in the bone marrow is best achieved by using ALK staining to highlight the rare tumour cells [3, 5]. 

Phenotypic differences between sample types have been described previously and mostly highlight the differences in intensity of T-cell antigen expression and CD30 and ALK positivity in the peripheral blood compared with bone marrow and lymph node [2, 3, 5–8]. It is presumed that the circulating cells represent the small cell population seen in the lymph nodes as they share similar phenotypic profiles [5, 8, 10]. Variability in intensity of expression may be partially attributed to the sensitivity of different techniques and may contribute to the effect of different phenotypes.

In our case, three phenotypically distinct populations were identified: the CD4/CD8 double-negative T-cell population seen in the peripheral blood and as a subpopulation in the lymph node and the CD8-positive small cell and the large strong CD30 populations seen in the bone marrow and lymph node. Although phenotypically different, all of these cell populations were demonstrated to have rearrangements of ALK by FISH analysis or ALK positivity by immunostaining and are therefore likely to be part of the same malignant clone.

Variability of antigen expression depending on sample type (blood, bone marrow, and lymph node) and leukaemic presentation complicate the diagnosis of this rare subtype of ALCL and by conventional microscopy, this variant is often mistaken for peripheral T-cell lymphoma, NOS [1, 2, 11, 12]. In this case, diagnosis was only made after extended immunohistochemistry and interphase FISH were performed. 

This case highlights the importance of the initial investigations and the routine inclusion of CD30 and ALK in all immunohistochemistry T-cell panels even when ALCL is not suspected. This would be especially important in the case of younger patients with constitutional symptoms and a leukaemic manifestation, when ALCL with a small cell pattern should be considered in the differential diagnoses.
